# Hyaluronic acid-enriched bilosomes: an approach to enhance ocular delivery of agomelatine via D-optimal design: formulation, *in vitro* characterization, and *in vivo* pharmacodynamic evaluation in rabbits

**DOI:** 10.1080/10717544.2022.2100513

**Published:** 2022-07-22

**Authors:** Asmaa Ashraf Nemr, Galal Mohamed El-Mahrouk, Hany Abdo Badie

**Affiliations:** Department of Pharmaceutics and Industrial Pharmacy, Faculty of Pharmacy, Cairo University, Egypt

**Keywords:** Agomelatine, glaucoma, bilosomes, hyaluronic acid, edge activator, bile salts, ocular delivery, D-optimal design, intraocular pressure, bioavailability

## Abstract

Agomelatine (AGO) is a dual-functional drug. It uses as an antidepressant when orally administrated and antiglaucomic when topically applied to the eye. This study aimed to formulate AGO into bilosomal vesicles for glaucoma treatment, as modern studies pointed out the effect of topical AGO on intraocular pressure for the treatment of glaucoma. A modified ethanol injection technique was used for the fabrication of AGO bilosomes according to a D-optimal design. Phosphatidylcholine (PC) to edge activator (EA) ratio, Hyaluronic acid percentage (HA%), and EA type were utilized as independent variables. The measured responses were percent entrapment efficiency (EE%), particle size (PS), polydispersity index, zeta potential, percentage of drug released after 2 h (Q_2h%_), and 24 h (Q_24h%_). The optimal bilosomal formula (OB), with the desirability of 0.814 and the composition of 2:1 PC: EA ratio, 0.26% w/v HA and sodium cholate as EA, was subjected to further *in vitro* characterizations and *in vivo* evaluation studies. The OB formula had EE% of 81.81 ± 0.23%, PS of 432.45 ± 0.85 nm, Q_2h%_ of 42.65 ± 0.52%, and Q_24h%_ of 75.14 ± 0.39%. It demonstrated a higher elasticity than their corresponding niosomes with a typical spherical shape of niosomes by using transmission electron microscope. It exhibited acceptable stability over three months. pH and Refractive index measurements together with the histopathological study ensured that the OB formula is safe for the eye and causes no ocular irritation or blurred vision. The OB formula showed superiority in the *in vivo* pharmacodynamics parameters over the AGO solution, so AGO-loaded bilosome could improve ocular delivery and the bioavailability of agomelatine.

## Introduction

The eye is a very unique organ of the body (Elazreg, 2014). Several diseases affect the eye. Glaucoma is a serious disease affecting the anterior chamber of the eye and may lead to blindness (Paul et al., 2010; Sun & Zhou, 2018; Emad Eldeeb et al., 2019; Wu et al., 2019). It occurs as a result of the elevation in the intraocular pressure (IOP) which causes progressive degeneration of the optic nerve, producing permanent loss of eyesight (Quigley, 2005; Weinreb et al., 2014; Kapetanakis et al., 2016). Consequently, glaucoma requires a lifetime treatment to be under control to prevent any dangerous progression (Mishra & Jain, 2014; Zeng et al., 2018).

Treatment of glaucoma involves adjusting the elevation of the IOP which occurs as a result of either increased production of aqueous humor or decreased drainage.

Melatonin is a hormone that is synthesized in different parts of the ocular tissues and has several receptors in the eye which are targets for melatonin action that plays a role in the reduction of IOP (Martínez-Águila et al., 2016). So, melatonin receptors agonist considers a novel approach for the treatment of glaucoma via lowering the IOP (Alarma-Estrany & Pintor, 2007; Pescosolido et al., 2015). Agomelatine is a dual-functional drug. It utilizes as an antidepressant when administrated orally, as it acts as a potent antagonist for the serotonin receptor-2C (De Bodinat et al., 2010). Also, it exerts a higher hypotonising activity when topically applied to the eye, as it acts as a potent agonist for the melatonin receptors (Martínez-Águila et al., 2016).

For several years, a thirty percent reduction in the IOP occurred after 30 days of the oral administration of agomelatine (Pescosolido et al., 2015). So, to enhance the hypotonising activity of agomelatine, either higher oral doses were required which in turn cause higher side effects or agomelatine must be topically applied to the ocular tissues to produce an effective IOP lowering effect at lower doses. To the author’s knowledge, only one researcher has ocularly delivered agomelatine as a mucoadhesive olaminosome nanovesicles to enhance its bioavailability and control its release (Abd-Elsalam & ElKasabgy, 2019).

Ocular drug delivery is exceedingly difficult as the eye has several protection mechanisms that reduce the absorption of the topically applied drug and lead to a short duration of action. These mechanisms include the drainage of tear fluid along with blinking of the eye leading to an around a 10-fold decline in the intraocular strength of the drug (Omerović & Vranić, 2020). Also, the limited corneal permeability and the passage of drugs via nasolacrimal play important roles to limit intraocular drug absorption (Sultana et al., 2006; Khalil et al., 2017; Fouda et al., 2018). Consequently, the bioavailability of topically applied drug solutions in the eye is not more than 1–5% (Järvinen et al., 1995; Ghate & Edelhauser, 2006; Elazreg, 2014).

Therefore, several nanovesicular drug delivery systems are utilized to enhance the ocular bioavailability, ocular contact time, residence time, and dosing regularity (Kakkar & Kaur, 2011; Elazreg, 2014) as liposomes, nanoemulsions, cubosomes, spanlastics, solid lipid nanoparticle, niosomes, and so forth.

Bilosomes (bile salts stabilized nanovesicles) are elastic vesicular carrier systems that resemble conventional niosomes consisting of a nonionic surfactant coupled with bile salts integrated within as edge activators (EAs) (Elnaggar et al., 2019; Rizwanullah et al., 2020; Zafar et al., 2021). EAs are nonionic surfactants used to impart elasticity and flexibility to the membrane of the conventional niosomes enabling them to squeeze themselves through pores smaller than their diameters. Hyaluronic acid (HA) is an amphiphilic compound; thus, the hydrophobic patch domain enhances the permeability of HA across the epithelium layer of the cornea and the hydrophilic patch domain enables HA to diffuse through the other corneal layer. Also, HA has been added to the developed bilosomes to enhance their bioadhesion properties to the corneal tissue resulting in prolonged corneal contact time, sustained ocular delivery, and hence, therapeutic efficacy (Yue et al., 2018; Fahmy et al., 2021).

The ability of bilosomes to deliver vaccine orally was studied and proved due to their ability to shield the drug from the bile salts and enzymes of the gastrointestinal tract due to the presence of bile salts in their composition (Aburahma, 2014; Jain et al., 2014; Al-Mahallawi et al., 2015). Bile salts are solubilizing and permeation-enhancing agents. They are widely used because of their biological compatibility and have no toxicity (Zafar et al., 2021). Also, the capability and the safety of the transdermal delivery of bilosomes were investigated and confirmed due to its nanosized vesicle and the elasticity that is required for transdermal delivery (Al-Mahallawi et al., 2015). Bilosomes have been also utilized for ocular delivery. The nanosized vesicles of bilosomes together with the presence of surfactants and bile salts in their compositions provide a promising attempt to assess them for ocular delivery (Mohsen et al., 2020).

So, this present study aims to encapsulate AGO inside bilosomal formulations that were prepared by the ethanol injection technique according to the D-optimal design to enhance its ocular permeation through the eye and to improve its ocular bioavailability followed by numerical optimization to select the optimal formula based on the desirability criterion.

## Materials and methods

### Materials

Agomelatine (AGO) was kindly supplied by Mash Premiere, Egypt. Hyaluronic acid (HA) (supplied as sodium hyaluronate, molecular weight 400–800 kdaltons) was purchased from Acros Organics, Belgium. L-a Phosphatidylcholine from egg yolk (_∼_60% by TLC) (PC), Sodium cholate (SC), Sodium deoxycholate (SDC), and Sodium taurocholate (STC) were purchased from Sigma-Aldrich® (Missouri, USA). Chloroform, methanol, disodium hydrogen phosphate, sodium chloride, and potassium dihydrogen orthophosphate were purchased from El-Nasr Pharmaceutical Chemicals Company, Cairo, Egypt. Spectra/Pore dialysis membrane (12,000–14,000 molecular weight cutoff) was purchased from Spectrum Laboratories Inc., (California, USA).

## Methods

### Experimental design

AGO-loaded ultradeformable bilosomes were prepared according to the D-optimal design using Design-Expert vii software (Stat-Ease, Inc., Minneapolis, MN, USA) to study the effect of different independent variables on vesicle properties (Nemr et al., 2021a). Phosphatidylcholine (PC) to edge activator (EA) weight ratio (PC: EA ratio), Hyaluronic acid percentage (HA%), and the type of the EA were chosen as independent variables to examine their effects on the studied dependent variables (Percentage of entrapment efficiency (EE%), Particle size (PS), Zeta potential (ZP), Polydispersity index (PDI), Percentage of drug released after 2 h (Q _2 h%_), and Percentage of drug released after 24 h (Q _24 h%_)).

According to the D-optimal design, 22 formulations (D1–D22) were prepared, its factors, their levels, and desirability constraints are shown in Table 1.

**Table 1.** D-optimal design factors, levels, and target constrains.

Independent variables (Factors)	Lowest level		Highest level
PC: EA ratio	2		4
HA%	0		0.5
Type of EA	SC	SDC	STC
Dependent variables (responses)		Constraints	
EE%		Maximize	
PS (nm)		Minimize	
Q_2h_		Minimize	
Q_24h_		Maximize	

EE%: percentage of entrapment efficiency; EA: edge activator; HA%: percentage of hyaluronic acid; PC: phosohatidylecholine; PS: particle size; Q_2h%_: percent of drug released after 2 h; Q_24h%_: percent of drug released after 24 h; SC: sodium cholate; SDC: sodium deoxycholate; STC: sodium taurocholate.

#### Construction of AGO-loaded bilosomes by a modified ethanol injection technique according to the D-optimal design

AGO-loaded bilosomes were prepared by a modified ethanol injection method (Kaur et al., 2012; Fahmy et al., 2021) Briefly, AGO and PC were dissolved in 4 mL of a 1:1 methanol: chloroform mixture. The organic mixture was added drop by drop into a hot (60 °C) aqueous phase consisting of distilled water in which the EA was previously dissolved. The mixture was stirred continuously on a hot magnetic stirrer at 800 rpm and 60 °C for 30 min (min) until complete evaporation of the organic solvent. Then, the final volume of the formulations was adjusted to 50 mL. HA was later sprinkled while stirring at 1000 rpm at room temperature until a homogenous dispersion was achieved. The formulations were kept in a refrigerator at 4 ± 2 °C overnight. All experiments were performed in triplicates, and the results were stated as mean ± *SD*.

##### In vitro characterization of AGO-loaded bilosomes

###### Determination of drug content and percentage entrapment efficiency (EE%)

For the drug content determination, one mL of AGO-loaded bilosomes was taken and placed in a 10 mL volumetric flask containing methanol, which was utilized to lyse the vesicles, followed by sonication to ensure complete disruption of all vesicles. Then, the clear solution was measured spectrophotometrically at *λ*_max_ of 267.4 nm using a UV/VIS spectrophotometer (model UV-1601 PC, Shimadzu, Kyoto, Japan).

The indirect method was utilized to calculate EE% of AGO, in which the unentrapped amount of AGO was measured and then subtracted from the drug content. One mL of AGO-loaded bilosomes was withdrawn and subjected to centrifugation at 22,000 rpm for 1 h using an ultra-cooling centrifuge (Sigma 3-30 KS, Sigma Laborzentrifugen GmbH, Germany) at 4 °C. Then, the supernatant was separated, diluted, and measured spectrophotometrically, by (Shimadzu, model UV-1601 PC, Kyoto, Japan) at *λ*_max_ = 267.4 nm (Fatouh et al., 2017a; Tawfik et al., 2020).

The percentage of the entrapped drug was calculated using the following formula:

(1) $$\mathrm{EE}\%=\frac{\mathrm{total}\;\mathrm{drug}\;\mathrm{content}\;(\mathrm{mg})-\;\mathrm{unentraped}\;\mathrm{amount}\;\mathrm{of}\;\mathrm{AGO}\;(\mathrm{mg})}{\mathrm{total}\;\mathrm{drug}\;\mathrm{content}\;(\mathrm{mg})}\times 100$$

###### Determination of PS, PDI, and ZP

Before measurements, each bilosomal formulation was subjected to proper dilution with deionized water to ensure the appropriate intensity of the light scattering. The mean PS, PDI, and ZP were measured using a Malvern Zetasizer Nano ZS at 25 °C (Malvern Instrument Ltd., Worcestershire, UK). PDI was an indicator of the homogeneity of the size distribution. The ZP measurement was carried out by observing the electrophoretic mobility of charged vesicles in an electrical field (Scognamiglio et al., 2013).

##### In vitro release studies and kinetic analysis of the release data

The *in vitro* dialyzing method was selected for conducting the *in vitro* release of AGO from the prepared bilosomal formulations. It was performed in a horizontal shaking water bath (GFL, Gesellschatt Laboratories, Berlin, Germany) adjusted at 60 rpm and 37 ± 0.5 °C (Abd-Elsalam & ElKasabgy, 2019). Briefly, 5 mL of AGO-loaded bilosomes and AGO solution in phosphate buffer saline (PBS) pH 7.4 (containing AGO equivalent to ∼1 mg) was added to a dialysis bag of the semipermeable membrane (12,000–14,000 molecular weight cut off) presoaked overnight in distilled water and placed in stoppered glass bottles containing 50 mL PBS with pH = 7.4. A 3 mL sample was withdrawn at a preset time interval of 0.5, 1, 2, 4, 6, 8,12, 24, and 48 h and substituted by an equal volume of fresh release medium to keep the volume constant and preserve sink condition (Nemr et al., 2021b). The percentage of drugs released at each time interval was determined spectrophotometrically at *λ*_max_ 267.6 nm. The release profiles were represented by plotting the cumulative percentages of drugs released against time (h).

The cumulative percentage of the drugs released was calculated using the following equation:

(2) $$Qn=\mathrm{Cn}\times \mathrm{Vr}+\sum_{i=1}^{n-1}Ci\times \mathrm{Vs}/\text{intial drug amount}$$

where:

Qn: Current cumulative percent of drugs released

Cn: The receptor medium current concentration at the nth sample

Vr: Receptor medium volume

Vs: Volume of each sample removed for analysis *n* − 1

$\sum_{i=1}^{n-1}Ci$: Summation of the previously measured concentrations.

Generally, the zero-order, first-order, second-order, and Higuchi’s square root equations were used for the analysis of the release data. A model with the highest coefficient (*R*^2^) value represents the suitable model.

##### Choosing the optimized bilosomal formula via the D-optimal design

Numerical optimization by Design-Expert® software version vii (Stat-Ease, Inc., Minneapolis, MN, USA) was utilized to choose the optimum formula regarding the significant factors and avoid the nonsignificant ones. The optimizing criterion was to boost EE% and Q_24h_%, in addition, diminish PS and Q_2h_%. The optimized bilosomal formula (OB), was the one with the highest desirability value (0.814). The OB formula is composed of PC: EA ratio 2:1, 0.26% w/v HA, and SC as EA type. It was subjected to further *in vitro* characterization.

#### Further *in vitro* characterization of optimized bilosomal formula

##### Measurement of the deformability index

The elasticity of the OB formula was determined by measuring its deformability index (DI) and compared to that of a similar niosomal formula that contained cholesterol instead of EA in its composition. Briefly, vesicles dispersion was extruded using an air compressor (Haug Kompressoren AG; Büchi Labortechnik AG, Flawil, Switzerland) adjusted at a pressure of 2.5 bar, through 200 nm pore size filters (Lei et al., 2013; Fahmy et al., 2021; Nemr et al., 2021b). The experiment was conducted in triplicate to take the average value.

The DI was determined using the following equation (Gupta et al., 2005):

(3) $${\mathrm{DI}\;=J(r_{v}/r_{p})}^{2}$$

where *J* is the weight of dispersion extruded in 10 min, *r_v_* is the size of vesicles after extrusion (nm), and *r_p_* is the pore size of the barrier (nm).

#### Morphological examination by transmission electron microscope

The morphological characteristics of the OB formula were examined using a transmission electron microscope (TEM) (Joel JEM 1400, Tokyo, Japan). Briefly, the dispersion was subjected to a proper dilution then a single drop was deposited on a carbon-coated copper grid and left to dry at room temperature. The examination was done at 80 kV(Fahmy et al., 2018; Aziz et al., 2019; Shahab et al., 2020).

##### Effect of terminal sterilization by gamma irradiation

The OB formula was exposed to sterilization using gamma irradiations arising from a 60 Co irradiator with a 10 kGy irradiation dose (Morsi et al., 2019) (National Center for Radiation Research and Technology, Nasr City, Egypt). The sterilized formula was re-assessed for their EE%, PS, and *in vitro* release studies using the same procedures utilized for the nonsterilized formula. The EE%, PS, Q_2h%_, and Q_24h%_ were compared before and after sterilization using a one-way ANOVA test. The *in vitro* release profile of the OB formula before and after sterilization was compared by calculating the similarity factor (*f*) according to the following equation (Moore & Flanner, 1996; Fahmy et al., 2018).

(4) $$f_{2}=50\;\log\{{\left[1+(\frac{1}{n}){\sum_{t=1}^{n}\left(R_{t}-T_{t}\right)}^{2}\right]}^{-0.5}\times 100\}$$

where:

*R_t_*: percentage of the drug released from OB before sterilization at time *t*.

*T_t_*: percentage of the drug released from OB after sterilization at time *t*.

##### Effect of short-term storage

The OB formula was stored in the refrigerator at 4–8 °C for three months in a sealed glass bottle. EE%, PS, Q_2h%_, and Q_24h%_ were compared for the OB formula using student t-test before and after storage to detect any possible changes that may occur during storage for these parameters (Fahmy et al., 2021).

##### Refractive index

The light refractive index (RI) for the OB formula was assessed using Hilger and Watts refractometer (model-46.17/63707, Hilger and Watts Ltd., London, UK), to ensure the safety of the applied formula on vision (Fouda et al., 2018; Fahmy et al., 2021).

##### Surface tension

The surface tension of the OB formula was estimated using the Du Nouy ring force tensiometer (model K-6, Krϋss GmbH, Hamburg, Germany) (Lee et al., 2012). The tensiometer was calibrated with distilled water and 30 mL of both water and OB formula were used for the test. The space among the dipped ring and liquid surface was set up at 4.5 mm. The surface tension of distilled water was 72 mN/m (Vicario-de-la-Torre et al., 2014; Fouda et al., 2018).

##### pH measurement

The pH of the OB formula was measured to confirm the safety of its ocular application. Measurement was carried out using a Jenway pH meter (model-3505, Bibby Scientific Ltd., Stone ST15 0SA, UK). The experiment was conducted in triplicate and the results were expressed as mean ± *SD* (Nemr et al., 2021a).

##### Differential scanning calorimetry

DSC analysis was performed for both pure drug (AGO) and the OB formula that was subjected to lyophilization using a Lyophilizer (Novalyphe-NL 500, Savant Instruments, Holbrook, NY) before measurement using a differential scanning calorimeter (DSC) (Shimadzu DSC 50; Kyoto, Japan)(Fahmy et al., 2019; Nemr et al., 2021a).

##### In vivo evaluation studies

The assessment of both the OB formula and the AGO solution in PBS for the possibility of ocular irritation, the measurement of IOP, and the potentiality of any histopathological alterations were performed on six healthy New Zealand male albino rabbits. The experimental procedures were pre-approved by the institutional review board of the Research Ethics Committee (REC) for the animal subject research at the Faculty of Pharmacy, Cairo University, Egypt (PI (3131)).

##### Ocular irritation study

The study was performed using six healthy New Zealand male albino rabbits weighing (2.5–3.0 kg) that were fed with food and water for 1 week to adapt to the environment before the study. Rabbits were subjected to both the OB formula in one eye and AGO solution (0.02% w/v of AGO) in the other eye to assess their potential for irritation according to the Draize test (Draize et al., 1944). The assessment Criteria of the Draize test are classified according to the scoring system from 0 (no irritation) to + 3 (highest irritation and redness) for the cornea, iris, and conjunctivae. A 100 μL of the OB formula was topically applied into the lower conjunctival cul-de-sac of the right eye, while the left eye was subjected to the AGO solution for comparison purposes. Subsequently, visual observations were carried out at 0 min, 30 min, 1 h, 2 h, 4 h, 6 h, 8 h, and 24 h.

##### IOP reduction study

The study design for measuring the IOP in rabbits with enhanced IOP was a simple, single-dose parallel design. Six male Albino rabbits (250–300 kg) contributed to the study. To enhance the IOP of the rabbits used, the rabbits were kept in dark for 24 h before performing the experiment (Okamoto et al., 2010). Before the application of the formulations, the IOP was measured and any animal showing any symptoms of irritation was not included in the study. A 10 μL of both formulations (OB and AGO solution containing ∼0.02%w/v AGO) were installed into the conjunctival sac of the right eye and the left eye, respectively. After installation, eyes were left open for 30 s to prevent the runoff of the drop. The IOP was measured using (SchiÖtz Tonometer (Rudolf Riester GmbH and Co. KG, Germany) twice at different time intervals of zero (pre-dose; IOP enhanced), 0.5, 1, 2, 4, 6, 8, and 24 h and average IOP was calculated.

##### Pharmacodynamic parameters

Four pharmacodynamic parameters were calculated using Kinetica VR software 2000 (Innaphase Corporation, Philadelphia, PA, USA). The studied pharmacodynamic parameters were the maximum % decrease in IOP (% dec IOP_max_), the time required to reach the maximum decrease (*T*_max_), mean residence time (MRT), together with the area under the percentage decrease in IOP against the time curve (AUC_0–24 h_) up to the last measured time point (24 h)(El-Mahrouk et al., 2009).

The % decrease in IOP was calculated according to the following equation (Ammar et al., 2009):

(5) $$\text{\% Decrease in IOP}=\frac{\text{IOP enhanced }-\text{ IOP treatment}}{\text{IOP enhanced }}\times 100$$

where:

IOP enhanced is the IOP at zero time and IOP treatment is the IOP of the treated rabbits’ eyes after instillation by time t.

##### Histopathological evaluation study

A histopathological examination study was carried out to check any possible ultrastructural changes in the rabbits’ eyes associated with the topical application of both OB formula and AGO solution in comparison with normal saline solution. Histopathological studies on the separated eyeballs were performed by a pathologist. Six rabbits (divided into two groups (A and B) with three rabbits in each group) were subjected to the investigation of the previously mentioned formulations. A 100 μL of the OB formula was topically applied three times daily for seven days into the lower conjunctival cul-de-sac of the right eye of the rabbits in both groups (A and B). While the left eye in groups A and B were subjected to AGO solution and normal saline solution, respectively for comparison purposes. On the seventh day, the treated rabbits were subjected to decapitation under light anesthesia. Next, the eyeballs were separated and washed with PBS (pH 7.4). For preservation purposes as well as to harden the fresh eye tissues, the separated eyeballs were directly fixed in 10% formol saline for 24 h. After 24 h, the fixed ocular tissues were then dehydrated using serial dilutions of ethyl alcohol. The dehydrated samples were then added to xylene and embedded in melted paraffin blocks at 56 °C for another day. Cross-sections from the paraffin blocks were cut and then stained using hematoxylin and eosin. Finally, the stained samples were examined microscopically under a light microscope (Leica Imaging Systems Ltd, Cambridge, England) fitted with a camera, Vic Tor, Model TK-C1380E (Japan) to detect any pathological changes (Baydoun et al., 2004).

## Results and discussions

### Factorial design analysis

Design-Expert software (V. vii, Stat-Ease Inc., Minneapolis, USA), was utilized for the statistical analysis of the results obtained from the D-optimal design, to assess the effect of the significant independent variables on the EE%, PS, PDI, ZP, Q_2h%_, and Q_24h%_, and to choose the OB formula according to the constraints that were listed in Table 1. As ZP and PDI values were acceptable throughout the entire design, these two responses were eliminated from the optimization process. To check the validity of the optimization process, the theoretical and practical values of the OB formula were compared and the results were shown in Table 2. 

**Table 2.** Theoretical and practical values for the OB formula.

Response	EE% ^a^	PS (nm) ^a^	Q_2h_ (%) ^a^	Q_24h_ (%) ^a^
Theoretical values	82.06	414.95	42.338	74.723
Practical values	81.81 ± 0.23	432.45 ± 0.85	42.652 ± 0.5196	75.138 ± 0.3891

a All data presented as mean ± *SD* (*n* = 3).

Abbreviations: EE%: percentage of entrapment efficiency; PS: particle size; Q_2h_%: percentage of drug released after 2 h; Q_24h_%: percentage of drug released after 24 h.

SPSS 20 software (SPSS Inc., Chicago, IL, USA) was used to apply a one-way ANOVA test to assess whether EE, PS, Q_2h%_, and Q_24h%_ were affected by gamma radiation and three-month storage. Also, it was used to apply two-way ANOVA (for % dec IOP_max_, AUC_0–24h_ to discover the significance of the formulations in the *in vivo* study.

The high similarity between the theoretical and the practical values indicates that the OB formula can be considered a promising formula for ocular AGO delivery with a higher bioavailability and lower side effects.

### Characterization of AGO-loaded bilosomes

#### Effect of formulation variables on EE% of AGO-loaded bilosomes

**Figure 1.**
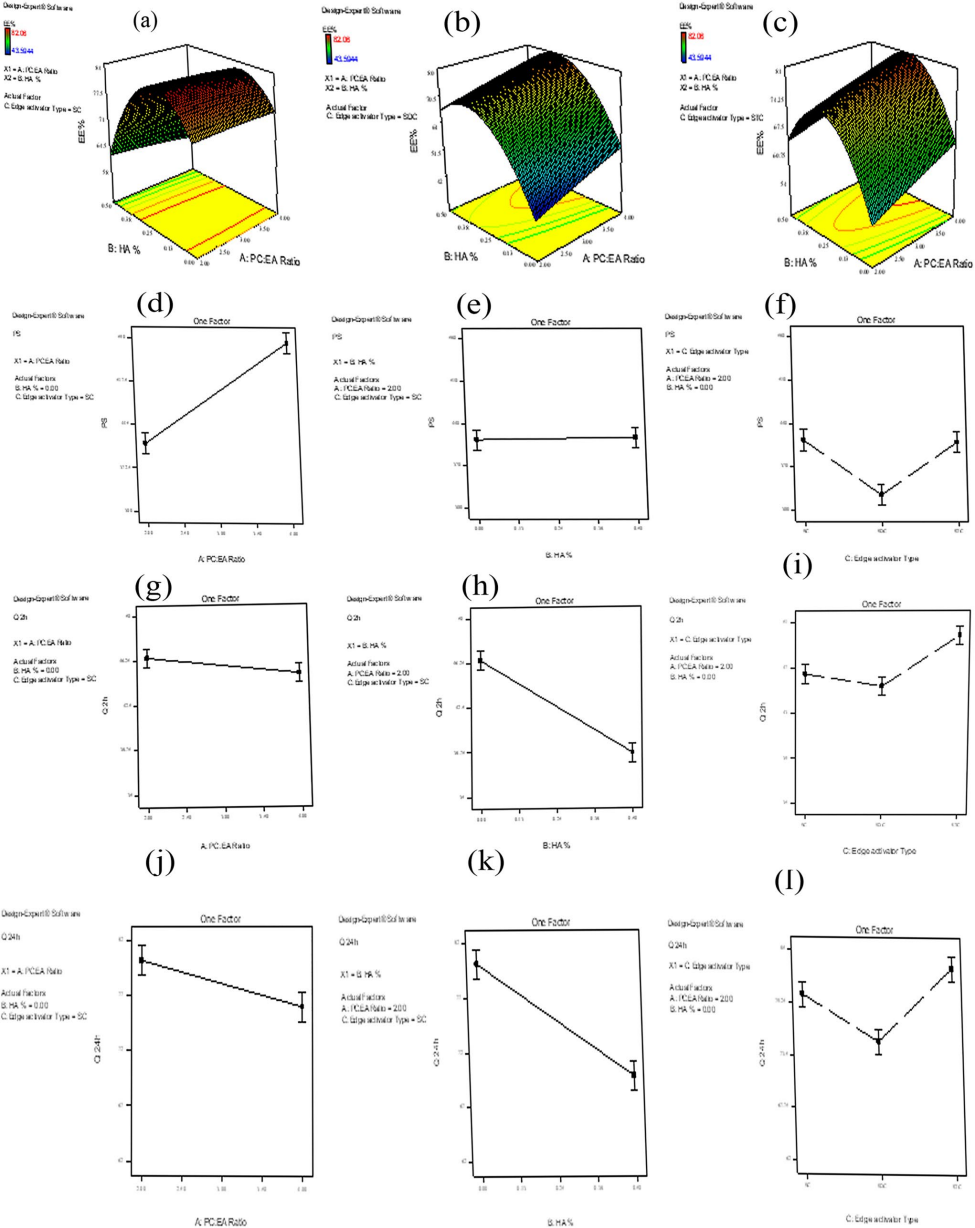
Response 3D plots for the effect of significant formulation variables on: percentage entrapment efficiency (EE%) (a–c), on particle size (d–f), on percentage of drug released after 2 h Q_2h%_ (g–i), and on percentage of drug released after 24 h Q_24h%_ (j–l) of agomelatine ultradeformable bilosomes.

The EE% of AGO-loaded bilosomes are presented in Table 3. The results showed that AGO was entrapped successfully inside all bilosomal formulae, where the EE% ranged from 43.59 to 82.06%. ANOVA analysis of the obtained results showed that all the studied independent variables had a significant effect on EE%. Considering the PC: EA ratio (*p* = .0001), increasing the ratio of PC from 2 to 4 resulted in a substantial increase in EE%. Also, it is observed that EE% was increased when HA% increased with the highest EE% at 0.26% (*p* = .0006) then EE% started to decrease. Regarding the type of EA, the highest EE% was obtained when using SC as EA (*p* < .0001). The effects of all factors on EE% of AGO-loaded bilosomes are illustrated graphically in Figure 1(a–c). The high EE% might be ascribed to the lipophilic nature of AGO that improves its entrapment inside the bilosomal formulations. Considering the PC: EA ratio, increasing the PC ratio results in increasing EE% as a result of increasing the lipophilicity of the vesicular system (Abdelbary & Aburahma, 2015). In addition, elevated levels of EA would lead to improving drug solubility in the dispersion medium due to the formation of micelles within the dispersion medium that prevents the entrapment of the drug inside the prepared vesicles, thus lowering the EE% (Niu et al., 2014). Solubilization of the drug happens when EA concentration reaches its critical micelle concentration (Van Den Bergh et al., 2001; Basha et al., 2013).

**Table 3.** Composition of the prepared AGO-loaded bilosomes and their *in vitro* characterization.

Formulation	PC: EA ratio	EA amount (mg)	EA type	HA%	EE% ^a^	PS (nm) ^a^	ZP (mV) ^a^	PDI ^a^	Q_2h_% ^a^	Q_24h_% ^a^
D1	4	100	STC	0.50	68.61 ± 1.41	565.45 ± 38.35	–42.20 ± 0.10	0.42 ± 0.11	40.28 ± 1.23	68.19 ± 2.83
D2	3.2	120	SC	0.50	56.55 ± 1.37	506.85 ± 6.35	–39.00 ± 0.60	0.24 ± 0.05	36.45 ± 2.45	65.98 ± 1.02
D3	4	100	SDC	0.50	74.03 ± 1.46	480.90 ± 14.30	–42.70 ± 1.60	0.30 ± 0.01	35.62 ± 1.03	62.37 ± 3.91
D4	2	166.7	SDC	0.00	43.59 ± 1.28	315.80 ± 0.20	–32.30 ± 4.00	0.34 ± 0.00	44.50 ± 4.23	76.69 ± 1.23
D5	2	166.7	STC	0.50	65.74 ± 0.83	448.10 ± 16.90	–44.20 ± 0.00	0.35 ± 0.03	41.85 ± 2.03	71.09 ± 2.04
D6	3.2	120	SC	0.50	62.08 ± 1.05	512.55 ± 0.65	–35.35 ± 0.25	0.25 ± 0.03	36.62 ± 1.80	66.43 ± 4.68
D7	3.6	110.6	SDC	0.25	77.58 ± 5.00	478.15 ± 15.05	–36.25 ± 0.65	0.24 ± 0.01	40.91 ± 2.05	65.36 ± 3.64
D8	2	166.7	SDC	0.25	68.93 ± 0.84	327.30 ± 9.50	–38.80 ± 0.50	0.50 ± 0.03	40.02 ± 4.10	68.33 ± 2.94
D9	4	100	SDC	0.00	50.64 ± 2.50	509.40 ± 49.40	–41.70 ± 2.80	0.34 ± 0.00	44.93 ± 3.89	68.39 ± 3.01
D10	2	166.7	SC	0.29	77.80 ± 0.96	426.15 ± 3.45	–28.05 ± 1.25	0.32 ± 0.01	41.92 ± 2.58	75.00 ± 1.04
D11	2.8	189.3	SC	2.80	75.67 ± 0.51	432.50 ± 6.20	–31.35 ± 0.05	0.43 ± 0.02	46.44 ± 1.04	78.31 ± 0.94
D12	2	166.7	SC	0.29	81.64 ± 0.05	492.05 ± 10.95	–42.00 ± 0.30	0.08 ± 0.13	41.92 ± 2.48	75.00 ± 2.71
D13	2	166.7	SDC	0.50	64.59 ± 2.29	306.00 ± 20.20	–36.25 ± 4.45	0.42 ± 0.00	38.89 ± 1.98	65.95 ± 3.14
D14	3.75	105.3	SC	0.00	73.53 ± 6.11	575.25 ± 18.75	–42.10 ± 0.20	0.47 ± 0.02	45.20 ± 1.65	75.16 ± 1.06
D15	4	100	SDC	0.00	55.45 ± 1.66	501.25 ± 17.75	–29.20 ± 1.10	0.34 ± 0.01	44.26 ± 1.23	71.44 ± 2.35
D16	3	125	STC	0.25	75.56 ± 2.66	482.95 ± 5.45	–35.05 ± 0.25	0.22 ± 0.07	45.13 ± 3.96	78.38 ± 1.46
D17	2.95	126.6	SC	0.25	80.22 ± 7.47	536.35 ± 4.95	–43.20 ± 1.90	0.19 ± 0.09	43.75 ± 0.92	75.98 ± 2.36
D18	3.84	103.3	STC	0.08	73.58 ± 3.49	561.30 ± 2.90	–35.20 ± 0.10	0.37 ± 0.02	47.20 ± 2.93	80.09 ± 0.92
D19	4	100	SC	0.21	82.06 ± 2.52	579.40 ± 3.10	–39.10 ± 0.60	0.23 ± 0.01	41.97 ± 1.58	70.63 ± 2.68
D20	2	166.7	STC	0.00	54.31 ± 0.00	406.10 ± 24.00	–26.10 ± 0.00	0.49 ± 0.03	49.28 ± 0.51	81.99 ± 1.05
D21	2	166.7	STC	0.50	62.98 ± 1.93	413.05 ± 2.75	–24.40 ± 0.00	0.28 ± 0.10	43.97 ± 2.93	71.97 ± 2.01
D22	2	166.7	STC	0.00	54.31 ± 0.00	413.60 ± 31.50	31.50 ± 0.00	0.49 ± 0.03	49.28 ± 1.95	81.99 ± 2.58

All formulae contain 10 mg drug and prepared in a 50 mL phosphate buffer saline (PBS) pH 7.4.

a Data presented as mean ± *SD* (*n* = 3).

Abbreviation: AGO: agomelatine; EE%: percentage of entrapment efficiency; EA: edge activator; HA%: percentage of hyaluronic acid; PC: phosphatidylcholine; PS: particle size; ZP: zeta potential; Q_2h%_: percentage of drug released after 2 h; Q_24h%_: percentage of drug released after 24 h; PDI: polydispersity index.

Regarding HA%, HA at high concentration decreased the EE%. This may be ascribed to its interaction and partial disruption of the vesicle membrane bilayer leading to the diffusion of the drug outside the vesicles (Fahmy et al., 2021). This finding was supported by (Wadhwa et al., 2010), who found that increasing the sodium hyaluronate ratio decreased the EE% of dorzolamide hydrochloride and timolol maleate in chitosan nanoparticles. Also, agree with (Fahmy et al., 2021) who reported that increasing HA% decreased the EE% during the ocular delivery of voriconazole inside elastosomal formulations. Concerning the EA type, it is obvious that bilosomes prepared using SC had significantly more entrapped AGO than other bilosomes. This could be attributed to the length of the alkyl chain affecting the HLB value of the EA. The lower the HLB value of EA, the higher will be the EE%. the HLB values of SC, SDC, and STC are 21.825, 21.925, and 39.175, respectively (Qiao et al., 2018).

#### Effect of formulation variables on PS of AGO-loaded bilosomes

Table 3 contains the PS values for all the developed bilosomal formulations that ranged from 306.20 to 579.40 nm. ANOVA analysis showed that Both PC: EA ratio and type of EA had a significant effect on PS (*p* < .0001 for both), however, HA% had no significant effect on PS (*p* = .7392). The effects of all factors on the PS of AGO-loaded bilosomes are illustrated graphically in Figure 1(d–f).

Regarding the PC: EA ratio, bilosomal formulae prepared with a higher PC ratio had higher PS than the corresponding ones that contained a lower PC ratio. This could be attributed to the bulk structure of PC. Also, increasing the PC ratio results in increasing lipophilicity of the prepared vesicles allowing them to entrap a higher amount of the lipophilic drug (Ahmed et al., 2020). Consequently, increasing both EE% and PS as there is a direct relation between EE% and PS (Hathout et al., 2007). Therefore, increasing the amount of EA results in producing vesicles of a smaller PS. This could be explained by bile salts are anionic surfactants, decreasing interfacial tension together with the subsequent formation of smaller vesicles (Dora et al., 2010; Aziz et al., 2019). Also, at a higher EA amount mixed micelle will be formed, which are smaller in their PS compared with vesicles (Al-Mahallawi et al., 2014). Considering the type of EA, SC-based bilosomes produced vesicles with larger PS than those prepared using STC and SDC. This could be attributed to the high negative charge on SC and higher ZP values of the vesicles formulated by using SC than those formulated with STC and SDC (Maldonado-Valderrama et al., 2011; Qiao et al., 2018). As vesicles of higher ZP values produced higher repulsion force between the charged vesicular bilayers with a subsequent increase in the spacing between them leading to the formation of larger vesicles (Aziz et al., 2019).

#### Zeta potential

The ZP values for all the developed bilosomal formulations are represented in Table 3. It ranged from −24.40 to −44.209 Mv, (Table 3). ZP is an indicator of the physical stability of the prepared bilosomal formulations, as it estimates the degree of repulsion between the adjacent vesicles. The highly negative ZP values could be attributed to the incorporation of the negatively charged HA that resulted in the massive adsorption on the surface of the prepared bilosomal formulations. This finding agrees with (Tran et al., 2014), who reported that increasing HA% led to inverse ZP values of the positively charged solid lipid nanoparticles. Additionally, these findings were similar to those (Fahmy et al., 2021) and (Wadhwa et al., 2010) upon the preparation of ultradeformable elastosomes of voriconazole and chitosan nanoparticles of dorzolamide, respectively.

#### Polydispersity index

PDI is a gauge for the homogeneity and uniformity of the prepared dispersions. Values close to 0 represent the homogeneity of the developed systems, while values close to 1 represent the heterogeneity of the systems (Zeisig et al., 1996; Ahmed et al., 2020). PDI values of the prepared bilosomal formulations varied from 0.083 to 0.5, (Table 3). These values indicate the homogeneity and uniformity of the developed systems (Younes et al., 2018; Fahmy et al., 2021) Nevertheless, ANOVA analysis showed that all the studied independent variables had no significant effect on PDI.

#### In vitro release and kinetic analysis of AGO-loaded bilosomes

**Figure 2.**
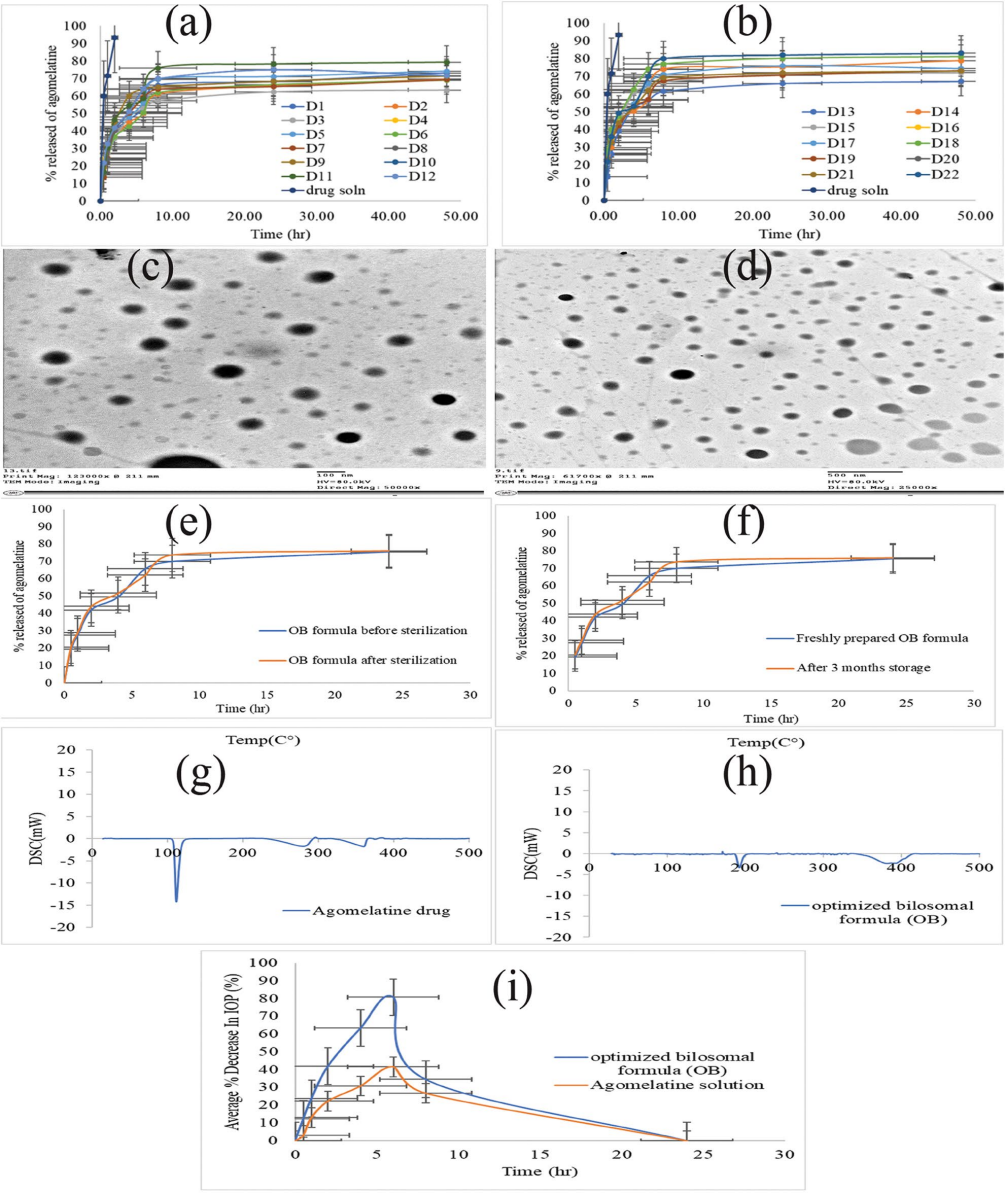
(a, b) the release profile of agomelatine-loaded bilosomes in comparsion with agomelatine solution, (c, d) the transmission electron microscope (TEM) image of the optimized bilosomal formula (OB), (e) the *in vitro* release profiles of the OB formula before and after gamma sterilization, (f) the *in vitro* release profiles of the freshly prepared and stored OB formula, (g, h) the DSC thermograms of pure agomelatine (g) and the lyophilized (OB) formula (h), (i) *in vivo* plot of the percentage decrease in IOP as a function of time response curve after ocular administration of OB formula and agomelatine solution in albino rabbits.

The release of AGO from the developed bilosomal formulations and AGO solution was evaluated in PBS 7.4, (Figure 2(a,b)). The percentage amount of AGO released from the prepared solution was approximately 93% after 2 h (Q_2h%_). This was an indication of the fast and uncontrolled release of AGO from the prepared solution

However, the release of AGO from all bilosomal formulations was slow and in a controlled manner, as the total percentage of AGO released after 2 h (Q_2h%_) did not exceed 45%. The release of all bilosomal formulations during 24 h (Q_24h%_) of the experiment showed a controlled release of AGO from the start to the end. At the end of the release, the total percentage of AGO released from all bilosomal formulations (Q_24h%_) ranged from 62.37 to 81.99%, while AGO solution released approximately 93% at the first 2 h. This ensured the proper encapsulation of AGO inside all bilosomal formulations. AGO release profile discriminators (Q_2h%_ and Q_24h%_) of AGO-loaded bilosomes are listed in Table 3.

Kinetic analysis of the release data was done according to zero-order, first-order, second-order, and Higuchi’s equations.

According to the value of the correlation coefficient (R^2^) obtained for zero-order, first-order, second-order, and Higuchi^’^s model. It was found that the release of AGO seemed to best fit Higuchi’s diffusion model.

#### The effect of formulation variables on Q_2h%_ and Q_24h%_ of the AGO-loaded bilosomes

The percent of AGO released from the developed bilosomal formulae after 2 h (Q_2h%_) ranged from 35.62 to 49.28%, while the percent of AGO released from the developed bilosomal formulae after 24 h (Q_24h%_) ranged from 62.37 to 81.99%. Conversely, the release of AGO from the prepared solution was almost complete after 2 h. The effects of all factors on Q_2h_% and Q_24h_% of AGO-loaded bilosomes are illustrated graphically in Figure 1(g–i) for Q_2h%_ and Figure 1(j–l) for Q_24h%_.

Statical analysis of the data showed that PC: EA ratio, HA%, and the type of EA, had a significant effect on both Q_2h%_ and Q_24h%_ (*p* = .0207 for PC: EA ratio, *p* < .0001 for both types of EAs and HA%). The release of AGO from the prepared bilosomes was inversely proposed with both the PC: EA ratio and HA%. The higher the PC ratio, the lower the Q_2h%_ and Q_24h%_ values. Also, increasing HA% from 0% to 0.5% results in slowing the release of AGO from the developed bilosomes. Regarding the type of EA, the highest Q_2h%_ and Q_24h%_ values were obtained with STC-based bilosomes, while SC and SDC-based bilosomes had slower release than those of STC-based ones. The slower the release of AGO from bilosomal formulations formulating with a higher PC: EA ratio could be explained in terms of lipophilicity. As AGO is a lipophilic drug, it prefers to retain inside lipophilic vesicles. This explained both its higher entrapment within bilosomal vesicles and its slower release toward the dissolution medium. The reciprocal relation between the entrapment efficiency and the percent of drug release was confirmed by (Abdelbary & Aburahma, 2015) who formulate lornoxicam as a proniosomal gel for the management of dental pain. Concerning HA%, a higher HA% results in slowing the release of AGO from the prepared bilosomal formulations. This could be explained by the increase in the viscosity that is imparted by increasing HA%. Concerning the type of EA, the higher release was observed with STC-based bilosomes. This could be explained by the lower EE% of the vesicles prepared using STC.

### Measurement of the elasticity of the optimized bilosomal formula

The elasticity of the OB formula was measured and compared with their corresponding niosomal formula that lacks the presence of EA in their composition. DI which is the measurement of the membrane elasticity was calculated for both OB and their corresponding niosomes. It was found to be equal to 16.67 ± 1.48 and 7.23 ± 0.18 for OB and conventional niosomes, respectively. Statical analysis of DI values has confirmed the superiority of the developed bilosomes over their corresponding niosomes (*p* = .024). This elasticity enables vesicles to squeeze themselves through pores of smaller size than their diameters and enhance their permeation (Trotta et al., 2002; Kakkar & Kaur, 2011; Fahmy et al., 2021).

### Morphology of the optimized bilosomal formula by TEM

TEM micrographs were used to determine the exterior morphology of the OB formula (Figure 2(c,d)). The TEM analysis demonstrates that the vesicles are identified with a spherical shape. Nevertheless, the PS obtained from Zetasizer was smaller than determined by the dynamic light scattering. This difference may be attributed to the dynamic light scattering measures the average PS (*Z*-average), while TEM measures the individual PS (Dahiya et al., 2018; Fahmy et al., 2021).

### Effect of terminal sterilization by gamma irradiation

Terminal sterilization is a method of sterilization performed to ensure the absence of microorganisms from the sterilized formulations. EE%, PS, and *in vitro* release analysis of the OB formula were reassessed after gamma sterilization. The results of EE%, PS, Q_2h%_, and Q_24h%_ before and after gamma sterilization were analyzed using a one-way ANOVA statistical test (at *p* = .05) and the results showed that there was no significant difference between them, (Table 5). The *in vitro* release profiles of the OB formula before and after sterilization are shown in Figure 2(e). Similarity factor (*f*) of the *in vitro* release data before and after gamma sterilization was calculated and it is found to be equal to 74.25, which is within the similarity range (50*-*100) (Moore & Flanner, 1996; Emad Eldeeb et al., 2019). From the obtained results, it could be concluded that gamma sterilization is an appropriate method for the sterilization of the AGO-loaded bilosomes causing no adverse effects.

**Table 4.** Values of EE%, PS, PDI, ZP, Q_2h_%, and Q_24h_% for Fresh and Stored OB formula.

Response	EE% ^a^	PS (nm) ^a^	PDI ^a^	ZP (mV) ^a^	Q_2h_ (%) ^a^	Q_24h_ (%) ^a^
Fresh OB formula	81.81 ± 0.23	432.45 ± 0.31	0.318 ± 0.017	–23.3 ± 0.3	42.652 ± 0.52	75.138 ± 0.389
Stored OB formula (3 months)	80.43 ± 0.45	441.7 ± 1.8	0.323 ± 0.005	–23.25 ± 0.6	43.4 ± 0.11	76.827 ± 0.118

a All data presented as mean ± *SD* (*n* = 3).

Abbreviations: EE%: percentage of entrapment efficiency; PS: particle size; PDI: polydispersity index; Q_2h_%: percentage of drug released after 2 h; Q_24h_%: percentage of drug released after 24 h; ZP: zeta potential.

**Table 5.** Results of the OB formula before and after gamma sterilization.

Response	EE% ^a^	PS (nm) ^a^	PDI ^a^	ZP (mV) ^a^	Q _2h_ (%) ^a^	Q_24h_ (%) ^a^
Before sterilization	81.81 ± 0.23	432.45 ± 0.301	0.318 ± 0.017	–23.3 ± 0.3	42.652 ± 0.5196	75.138 ± 0.3891
After sterilization	97.17 ± 0.5	434 ± 4.5	0.3315 ± 0.0015	–23.2 ± 0.2	44.43 ± 0.00	76.872 ± 0.1174

a All data presented as mean ± *SD* (*n* = 3).

Abbreviations: EE%: percentage of entrapment efficiency; PS: particle size; PDI: polydispersity index; Q_2h_%: percentage of drug released after 2 h; Q_24h_%: percentage of drug released after 24 h; ZP: zeta potential.

## Effect of short-term storage

After three months of storage for the OB formula, there was no change in the physical appearance of the formula. The results of EE%, PS, PDI, ZP, Q_2h%_, and Q_24h%_ for both freshly prepared OB formula and stored one are listed in Table 4. The *in vitro* release profiles of the freshly prepared and stored OB formula are shown in Figure 2(f). There was no significant difference in EE%, PS, PDI, ZP, Q_2h%_, and Q_24h%_ (*p* > .05) of the stored sample when compared by the fresh formula by one-way ANOVA test. As there was no change in the physical appearance of the OB formula after three-month storage. This suggests the high stability of the OB formula which could be attributed to the anionic nature of SC that imparts a high negative charge to the formula (Abdelbary et al., 2016). Also, the presence of PC together with SC led to the formation of the mixed micelle (Tan et al., 2013).

### Refractive index

The RI of the OB formula was found to be 1.3236, which is within the acceptable range. RI is a pointer for the patient’s discomfort due to the distorted vision after administration of ophthalmic preparations. The acceptable value of the RI is less than 1.5 (Ammar et al., 2009; Fouda et al., 2018; Fahmy et al., 2021). The RI of the OB formula was found to be 1.3236 which is within the acceptable range, causing no discomfort.

#### Surface tension

Surface tension is an important parameter for ocular formulations as it affects the penetration of the drug through the cornea. The surface tension of tear film is about 44 mN/m (Tiffany et al., 1989). The closer the value of the ocular formulations to that of the tear film, the longer the time it stays on the corneal surface (Doshi & Xu, 2009). The surface tension of the OB formula was found to be 48.7 mN/m. It may be due to the presence of the surfactant and phospholipid in its composition (Vicario-de-la-Torre et al., 2014; Fouda et al., 2018).

#### pH measurement

pH value of the OB formula was found to be 7.23 ± 0.19. This pH value is compatible with that of the tear fluid (pH = 7.4) (Kuno & Fujii, 2011) and does not irritate the eye after administration.

### Differential scanning calorimetry

DSC study is used to detect the possible interaction between the drug and the used excipients and to assess the crystallinity of the drug within the developed formula. The DSC thermograms of pure AGO and the lyophilized OB formula were depicted in Figure 2(g,h). AGO shows a sharp endothermic peak at 102 °C, corresponding to its melting point (Fatouh et al., 2017b; Tawfik et al., 2020), which describes the crystalline nature of AGO. The disappearance of this characteristic peak in the lyophilized OB thermogram signifies the existence of AGO in the amorphous state within the OB formula.

#### In vivo evaluation of AGO-loaded bilosomes

##### Ocular irritation Draize test

The Draize test was performed to detect the ocular tolerability and safety of the topically applied OB formula against the AGO solution. The OB formula as well as AGO solution showed no visual irritation on the rabbit’s eye and scored zero on the Draize scale during the whole study. So, it could be deduced that the OB formula was nonirritant, safe, and well-tolerated by the rabbits’ eyes. From these results, it could be concluded that the cornea, conjunctiva, and iris were all free from any inflammation.

#### Pharmacodynamic study for the evaluation of the IOP effect of the optimized bilosomal formula in rabbits with enhanced IOP

Figure 2(i) demonstrates the effect of the OB formula on the % decrease in IOP for the six male albino rabbits at predetermined time intervals in comparison with the AGO solution (0.02% w/v). Four pharmacodynamic parameters (% decrease in IOP_max_, *T*_max_, MRT, and AUC_0–24 h_) were obtained for both OB formula and AGO solution by using Kinetica VR software. The *in vivo* results revealed that the AGO solution reached its maximum % decrease in IOP_max_ after 6 h with a value of 35.92% ± 2.8, then, % decrease in IOP started to decrease gradually till returned to its normal values at the end of the study (24 h). Conversely, the OB formula showed a maximum % decrease in IOP_max_ with a value of 82.682 ± 3.97% after 6 h from the ocular installation which was significantly higher than that with the AGO solution. Both OB formula and AGO solution reached their maximum % decrease in IOP_max_ after 6 h (*T*_max_), but it is obvious that the IOP_max_ achieved by the OB formula is much higher than that obtained by the AGO solution (82.682%, 35.92%, respectively). This ensures the superiority of the OB formula over the AGO solution. The MRT was 13.36 ± 5.19 h and 7.52 ± 1.99 h for the OB formula and AGO solution, respectively. Wilcoxon signed-rank sum test application to MRT values yielded a *Z* value of −2.023. This *Z* value yielded an asymptotic significance of 0.043 which means that the OB formula resulted in significantly higher MRT than the AGO solution. This increased MRT ensured the sustainment of the drug release. Regarding the area under the % decrease in the IOP response curve (AUC_0–24h_), it was statistically significantly higher for the OB formula than that of the AGO solution (409.40%.h ± 37.03, 181.30%.h ± 15.01, respectively). This ensured the higher bioavailability of the OB formula than the AGO solution.

The higher bioavailability of the OB formula over the AGO solution could be attributed to the bilosomal components (nonionic surfactants (SC) and PC) which act as penetration enhancers and help in the diffusion of the drug to the cornea (Ammar et al., 2011; Khatoon et al., 2017).

Together with the presence of HA as one of the formulation components which enhances the interaction with the corneal surface, leading to resistance of the formulae to the effect of blinking and tear film turnover (Hao et al., 2002; Sayed et al., 2020; Fahmy et al., 2021)

Moreover, the inclusion of AGO inside bilosomal vesicles protects it from the degradation by the metabolic enzymes existing in the tears and the corneal epithelial surface consequently, enhancing its ocular bioavailability (Dhangar et al., 2014; Li et al., 2014).

#### Histopathological evaluation study (safety)

**Figure 3.**
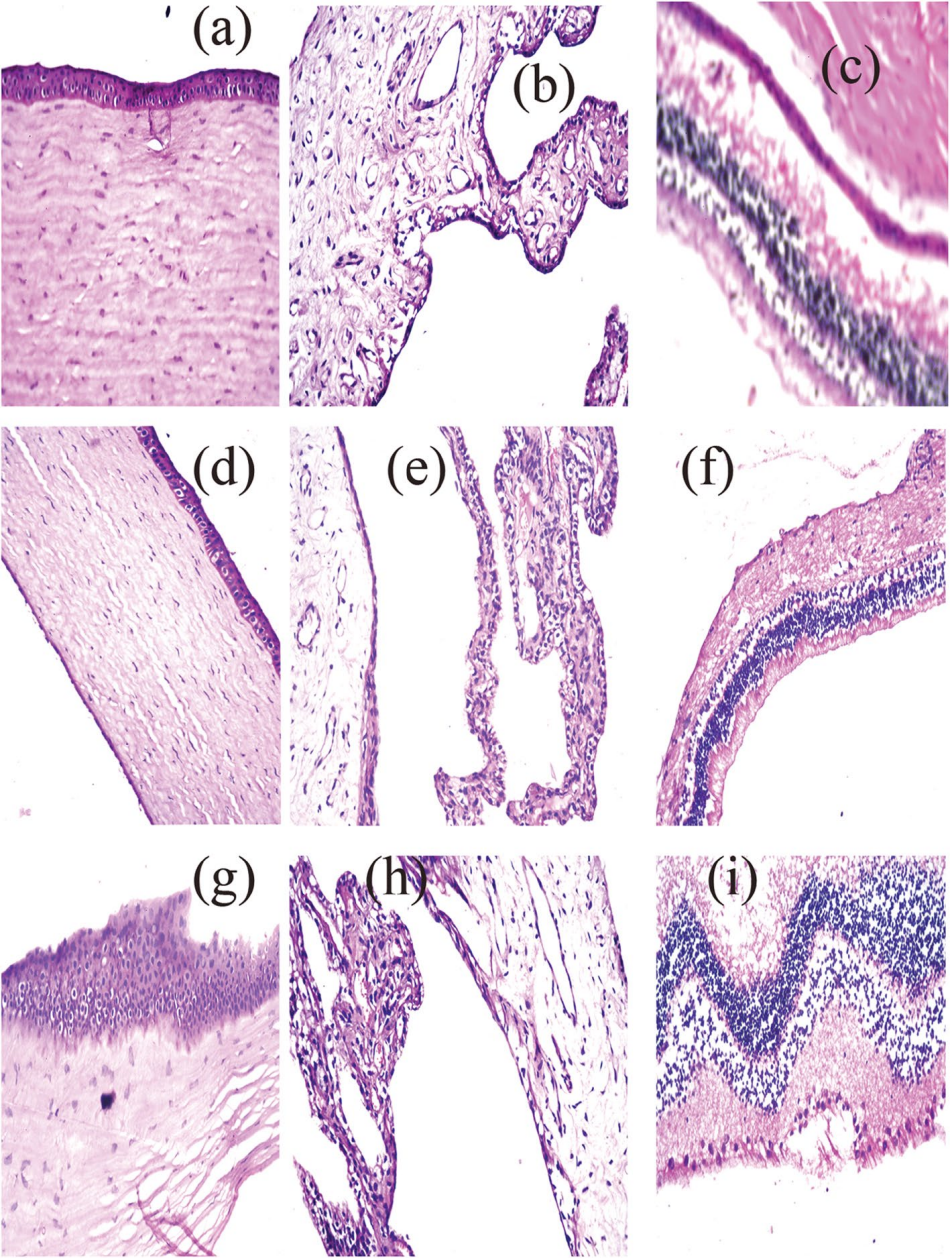
represt photomicrographs of histopathological sections of rabbit cornea, iris, retina, choroid, and sclera treated with: (a–c) negative control, (d–f) OB formula formula, and (g–i) agomelatine solution.

A histopathological examination study was very important to ensure the safety of the OB formula for the ocular application. The examination of photomicrographs showed that the exposure of rabbit eyes to normal saline as a negative control (Figure 3(a–c)) and OB formula (Figure 3(d–f)) did not cause any sign of irritation. All cornea, iris, retina, choroid, and sclera showed no histopathological alteration and maintained the normal histological structure of the lining epithelium and the underlying stroma. While the photomicrographs of the AGO solution showed cornea with focal stratification in the lining epithelium (Figure 3(g)), iris, choroid, and sclera with no histopathological alteration as recorded in Figure 3(h), and retina with focal inflammatory cells infiltration at the peripheral zone (Figure 3(i)). This confirms the superiority of the OB formula over the AGO solution for the ocular application.

Consequently, it can be concluded that the OB formula can be safely applied to the eye.

## Conclusion

In this study, AGO was successfully entrapped inside all bilosomal formulations that were prepared by ethanol injection technique, according to a 3-factor D-optimal design. The suggested OB formula according to the desirability criterion composed of PC: EA ratio 2:1, 0.26% w/v HA, and SC as EA type, exhibited higher EE% (81.81 ± 0.23%) and Q_24h%_ (75.138 ± 0.389%) together with smaller PS (432.45 ± 0.85 nm) and Q_2h%_ (42.652 ± 0.5196%). There was no significant difference between the theoretical and practical values this ensured the validity of the design. The elasticity test confirmed the superiority of the OB formula over their corresponding niosomes. TEM visualization demonstrates the vesicles with a perfect spherical shape. The OB formula showed acceptable stability over three months of storage in a refrigerator at 4-8 °C. Gamma sterilization of the OB formula causes no significant effect on the *in vitro* characterization, so it is an appropriate method for the sterilization of AGO-loaded bilosomes causing no adverse effects. The ocular irritation test ensured the safety of the ocular application of the OB formula which was expected from the results of pH and RI. Histopathological evaluation test revealed the safety of the ocular administration. In the *in vivo* study, the measured pharmacodynamic parameters for the OB formula (% decrease in IOP_max_, *T*_max_, MRT, and AUC_0–24h_) compared to the AGO solution were: 82.682%±3.97, 6 h, 7.52 ± 1.99 h, and 409.40%.h ± 37.03 versus 35.92%±2.8, 6 h, 13.36 ± 5.19 h, 181.30%.h ± 15.01, respectively). These results showed that the OB formula could result in a higher reduction in IOP and significantly increase AGO bioavailability compared to the AGO solution.

Consequently, it can be concluded that encapsulation of AGO inside bilosomal formulations improves the IOP reducing effect of AGO and enhanced its bioavailability and it is very promising for the treatment of glaucoma with a higher bioavailability and lower side effects.
